# Perspectives on prevention of type 1 diabetes and heterogeneities

**DOI:** 10.1007/s00125-025-06512-5

**Published:** 2025-08-06

**Authors:** Lars C. Stene

**Affiliations:** 1 Department of Chronic Diseases, Norwegian Institute of Public Health, Oslo, Norway; 2 Oslo Diabetes Research Centre, Norwegian Institute of Public Health, Oslo, Norway

**Keywords:** Heterogeneity, Human, Prevention, Randomised trials, Review, Type 1 diabetes, Uncertainty

## Abstract

Preventing type 1 diabetes remains a significant challenge but ongoing efforts are bringing us closer to this goal. This article discusses some implications of heterogeneity and chance in relation to prevention of type 1 diabetes, particularly regarding interpretation of evidence and planning of future trials. Using simulations, I illustrate uncertainties in efficacy estimates in prevention trials with time-to-event endpoints, using the TN10 teplizumab trial as an example. I emphasise that risk heterogeneity does not equate to treatment effect heterogeneity. When factors modifying efficacy are taken into account, robust identification of treatment effect heterogeneity may require sample sizes approximately four times larger than those needed to determine overall efficacy in certain scenarios. Efficiency of prevention trials can be increased using 2 × 2 factorial designs investigating two treatment options. I also simulate statistical power in exploratory studies involving multiple testing with different strategies for handling potential type 1 diabetes endotypes. If endotypes are defined as subtypes of type 1 diabetes-related phenotypes with at least partially unique risk factors, it becomes clear that we should aim to discover actionable aetiological factors that are not endotype-specific. Subjective judgements and pragmatism will influence whether and how a prevention trial is planned. Current approaches target high-risk individuals, which reduces the required number of trial participants but increases the cost of identifying trial participants. A prevention trial targeting infants in the general population with a multivalent antiviral vaccine will likely need over 50,000 participants, depending on circumstances and assumptions. While such a trial is conceivable, it would demand robust safety data before initiation.

**Graphical Abstract:**

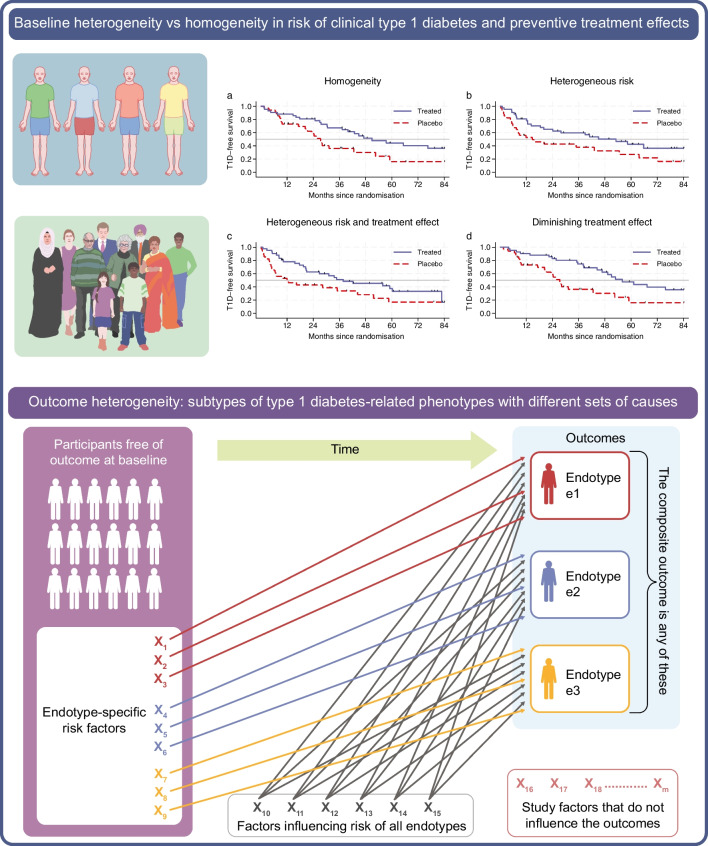

**Supplementary Information:**

The online version contains peer-reviewed but unedited supplementary material available at 10.1007/s00125-025-06512-5.

## Introduction

**Fig. 1:**
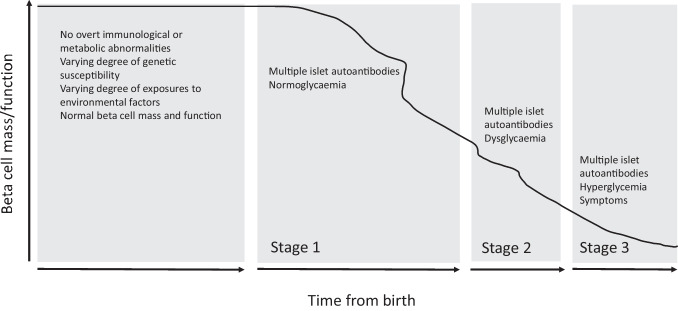
Natural history of type 1 diabetes and proposed stages of preclinical disease. Reprinted with permission from Springer Nature from [6]

Type 1 diabetes is an immune-mediated disease that results in a lifelong need for exogenous insulin and a high burden of disease for those affected and the society. Important attempts to cure or prevent the disease created hope several decades ago but largely failed [1]. The first large-scale prevention trials completed over 20 years ago demonstrated an impressive ability to identify high-risk individuals [2]. Since then, our understanding of the natural history and mechanisms underlying autoimmune type 1 diabetes in humans has been refined [3–5]. A staging scheme for preclinical disease has been proposed, based primarily on the presence of islet autoantibodies and dysglycaemia (Fig. 1).

Several mechanistically oriented immunomodulation trials attempting to preserve endogenous insulin production after diagnosis of type 1 diabetes have failed, while a few have showed promising initial results [7, 8]. It is hoped that effects can be extrapolated to earlier stages of the disease. Most prevention trials have attempted to slow the progression from islet autoimmunity to clinical type 1 diabetes without success [7, 9, 10]. A remarkable exception was the delayed progression from islet autoimmunity with dysglycaemia (stage 2) to clinical type 1 diabetes (stage 3) using a T cell-directed monoclonal antibody (teplizumab) given as a single, 14-consecutive-day course [11], now approved for use in the USA. Recent reviews have considered interventions with immunotherapy [4, 12–14]. The difficulty of preventing type 1 diabetes has been attributed to disease heterogeneity and incomplete knowledge of the aetiopathogenesis. Only one large-scale primary prevention trial has been completed [15], while more are underway. It is possible that early intervention before clonal expansion of autoreactive lymphocytes may have a greater chance of success.

Rather than review all previous prevention studies and recent immunological advances, the aim of the present review is to present some perspectives that hopefully will complement ongoing efforts and other reviews on the prevention of type 1 diabetes. I will discuss various kinds of heterogeneity, the role of chance, uncertainty in efficacy estimates, statistical power and implications for planning future trials.

## What is prevention?

*Most useful interventions or therapies change, for the better, the chance of a favourable outcome - change it from a smaller chance to a larger chance*

John W. Tukey [16]

While the goal is to completely prevent everybody from developing type 1 diabetes, this may not be realistic. In line with the citation above and modern causal inference literature, a useful operational definition of a preventive intervention is one that reduces the incidence of disease in the target population [17–19]. This definition includes delayed onset. Even in prevention trials targeting low-risk individuals, we can never know whether an individual in the intervention group who is free from disease at the end of follow-up will develop type 1 diabetes shortly after. Individual treatment effects can never be observed [16, 17, 20].

Furthermore, the staging criteria for type 1 diabetes not only redefine diabetes (defined by hyperglycaemia) but complicate the terminology for prevention since one cannot prevent something (type 1 diabetes) one already has [21]. Interception is perhaps a better term for reducing the rate of progression from islet autoimmunity to clinical type 1 diabetes. Reduction of the incidence of islet autoimmunity has been referred to as primary prevention.

## Judging trial evidence

Randomised trials are the gold standard for evaluating effects of interventions but may still have biases and random variation. Extensive methodological literature helps to increase the transparency and rigor of the process of judging trial evidence for application but judgements are required [22]. Regulatory bodies evaluate trial evidence in terms of efficacy and safety before approving a drug for application, usually based on several trials [23]. In the case of teplizumab, the treatment had been given to individuals newly diagnosed with (stage 3) type 1 diabetes in five previous trials with the aim of preserving endogenous insulin production and providing data on safety [24]. While the latter is overall reassuring, the time to stage 3 type 1 diabetes remains the gold standard outcome for trials demonstrating efficacy in preventing or delaying stage 3 type 1 diabetes. In the TrialNet Anti-CD3 Prevention Trial (TN10), teplizumab showed efficacy in reducing the rate of progression from stage 2 to stage 3 type 1 diabetes. The study was well-designed and well-conducted but it is a single randomised placebo-controlled phase II trial with 76 participants in total, aged 8–45 years, with confirmed multiple islet autoimmunity and dysglycaemia [11]. While original sample size calculations based on 50% assumed efficacy indicated a need for 144 participants, slower than expected recruitment led investigators to change to 60% assumed efficacy (HR 0.4) and calculations indicated a need for a total of 40 events for 80% power. The duration of follow-up varied from less than 6 months to more than 5 years, with a median of 2 years. The observed HR was 0.41 (95% CI 0.22, 0.78). The US Food and Drug Administration (FDA) approval raises practical questions for future trials if teplizumab is considered to be the established best treatment:

- is it unethical to attempt replication of the TN10 trial?
- should any new trial aiming to delay progression from stage 2 to 3 type 1 diabetes use teplizumab as a comparator rather than a placebo?
- should new trials targeting individuals without islet autoimmunity or individuals with stage 1 diabetes offer treatment with teplizumab, free of charge, to those who later progress to stage 2?

The latter two questions in particular have immense implications for future trials in terms of cost and required infrastructure. Below, I elaborate on how chance and heterogeneity may produce uncertainty in estimates from trials and observational human studies. I illustrate the points with simulations (‘virtual trials’) imitating the TN10 trial under different scenarios.

### Uncertainty in effect estimates from trials

**Fig. 2:**
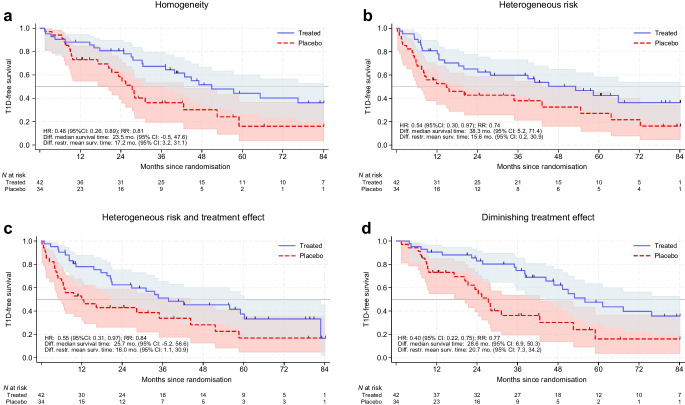
Simulation of uncertainty and heterogeneity in a prevention trial resembling the TN10 teplizumab trial. Kaplan–Meier survival function (with 95% CIs) for data simulated to imitate the TN10 teplizumab trial to prevent progression from stage 2 to stage 3 type 1 diabetes, with total *n*=76 and different underlying scenarios of heterogeneous participants. (**a**) Completely homogeneous (all participants have identical underlying risk and treatment effect). (**b**) Heterogeneity among participants in risk of progression but all individuals have equal treatment effect HR. (**c**) Underlying heterogeneity in both risk of progression and preventive treatment effect HR (average 33% efficacy in the lowest 15% of the distribution and 74% efficacy in the upper 15% of the distribution of the unobserved effect modifier). (**d**) The underlying preventive treatment effect is initially strong (HR 0.15) but diminishes over time to approximately 0.7 at 84 months. Individual trial participants are homogenous with respect to risk of type 1 diabetes and preventive treatment effect. The HR represents the observed (unadjusted) HR estimated from the simulated data using Cox regression. RR, risk ratio, represents the ratio of incidence proportions. Thin, black tick marks on the survival curves indicate censoring events. The 95% CI for the difference in median survival time was obtained using bootstrapping. The restricted mean survival time at 84 months of follow-up is explained in the ESM Methods text, together with additional details of the simulated scenarios. Large-sample simulations of these scenarios are shown in ESM Fig. 1. Diff., difference; mo., months; restr., restricted; surv., survival; T1D, type 1 diabetes

Figure 2 shows simulations of a trial similar to the TN10 trial (in which teplizumab delayed time from stage 2 to stage 3 type 1 diabetes [11]) under four different scenarios. In Fig. 2a, all individuals have equal baseline risk and treatment effect. In Fig. 2b, the treatment effect is the same for all but baseline risk differs between individuals. In Fig. 2c, both the baseline risk and the treatment effect differ between individuals. In Fig. 2d, all individuals have equal baseline risk and treatment effect, but treatment effect diminishes over time. One should interpret the shape of Kaplan–Meier curves with caution. Note, for instance that in the presence of unobserved heterogeneity in risk (Fig. 2b), the treatment effect will also appear to diminish over time even if the true effect is constant over time; see electronic supplementary material (ESM) Fig. 1 and ESM Methods for details. While an infinite number of scenarios are possible, these four simplified scenarios show that different underlying scenarios of heterogeneity can produce similar results. It is difficult to differentiate between these scenarios using trial data because trials are typically underpowered to identify treatment heterogeneity or variation over time. Point estimates of efficacy inform prospective treatment recipients, physicians and payers but they must be interpreted considering the (im)precision. The 95% CIs around point measures of average effect are quite wide regardless of heterogeneity.

#### Different measures of effect

Different measures of preventive effect, such as HR, RR and difference in median survival time, each have unique qualities. While HRs are commonly used for sample size calculations and estimating efficacy and statistical significance, the difference in median survival time has been used to inform potential recipients of the intervention in the case of teplizumab. However, this metric is based on a narrow part of the survival curve and typically has a wide CI.

In the scenario where all individuals have equal baseline risk and treatment effect, the point estimate for difference in median survival time is 23.5 months, and the 95% CI ranges from −0.5 to 48 months despite underlying homogeneity of the preventive treatment effect. Additional simulations of the scenario illustrated in Fig. 2a, but increasing the sample size to 200, still gave quite wide CIs for the difference in median survival time (8 to 46), while with *n*=650 the 95% CI was (16.8 to 36.4). The resources required to replicate the trial with such a sample size are considerable.

The difference in restricted mean survival time is an alternative measure based on the whole survival curve that can be estimated even if estimated survival is less than 50% (for further explanation, see e.g. [25] and ESM Methods). The estimated difference in mean restricted survival time in Fig. 2a was 17.2 months with a 95% CI from 3.2 to 31, which is still quite wide. In addition to possible treatment effect heterogeneity, even the magnitude of the average treatment effect remains imprecisely estimated. Therefore, information provided to individuals considering the intervention, as well as other stakeholders, should account for this uncertainty.

#### Bias in randomised trials

Trials are usually initiated based on several lines of promising preclinical evidence. There are, however, many intricate ways in which such data are not robust, as explained for instance in [26], and exemplified by von Herrath et al for the NOD mouse model of autoimmune diabetes [27]. Additionally, if the mechanistic and other preclinical evidence is robust, well-designed trials may still produce biased results, as noted for instance by Stuart Pocock in his influential book on randomised trials from 1983 ([28], p. 242). Even for truly efficacious interventions, the efficacy may be overestimated from single, small trials. The statistical power of a trial is always evaluated against an unknown true magnitude of effect. If investigators overestimate the efficacy, the trial will be underpowered. Because of the cost and time required for trials, many are underpowered. In this situation, the phenomenon of the winner’s curse can occur. After many trials fail to show significance, the first trial to demonstrate statistical significance may overestimate the magnitude of effect, even when there is a true effect [29]. Both theory and trial data (e.g. from rheumatology) show that efficacy estimated from larger phase III trials is lower than that estimated from smaller phase II trials [30–32]. Although this may not necessarily apply to the TN10 trial, this general phenomenon contributes to the uncertainty of the efficacy estimate and underscores the importance of trial replication before any intervention is widely adopted.

## Heterogeneity, chance and multiplicity

Statistical associations can result from causal effects, chance and bias. The relevant question is how much of each. In addition to creating spurious association, chance can create imprecision in causal effect estimates and of heterogeneity.

### Heterogeneity, heterogeneity and heterogeneity

*Describing aspects of biology as ‘heterogeneous’ often has a negative connotation. It is a term that is used when we do not understand a measured or observed aspect of disease or when we need to explain data that are not consistent*

Battaglia et al [33]

Heterogeneity is everywhere we look for it. No two humans are alike, including those with type 1 diabetes. We must accept this and still make pragmatic simplifications to advance. It is essential to distinguish between different forms of heterogeneity (see text box: Types of heterogeneity).


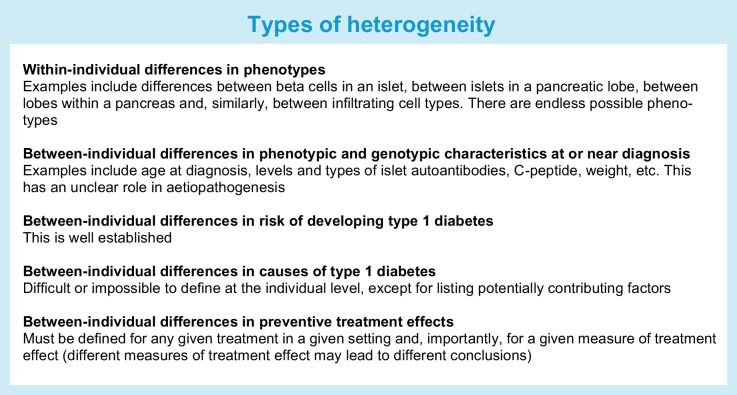


The challenge lies in identifying relevant sources and types of heterogeneity. What variation is part of a spectrum, and what constitutes a distinct entity? Data can be used for description, prediction or causal inference. Description of heterogeneity may offer limited insights into causality. For example, inter-individual differences in islets and immune cells can only be used to generate hypotheses about disease mechanisms, and phenotypes observed at or near clinical diagnosis may not provide much information about what happened during initiation of the disease process.

Heterogeneity in risk of type 1 diabetes is well established. The true risk of developing type 1 diabetes is unknown but we can stratify people based on combinations of established risk factors. The observed risk is simply the average risk within a given sample, as for the scenario shown in Fig. 2b.

What caused type 1 diabetes in an individual cannot be determined with certainty, and to predict it before the fact is even more difficult. It is possible, however, to identify factors potentially contributing to the risk of type 1 diabetes. Such factors could be necessary, sufficient or both, but typically they are neither. Whether individuals with type 1 diabetes observed to have been exposed to different causes or risk factors should be labelled as different (endo)types, is an ongoing debate [33, 34]. Monogenic autoimmune syndromes can serve as an example: despite the homogeneity of their causes, the phenotypic expression can be variable [35–37]. In Fig. 2a, time to type 1 diabetes varied from a few weeks to over 7 years, even when all individuals have the same underlying risk. This underscores the notion that phenotypic heterogeneity should not automatically be interpreted as evidence for aetiological heterogeneity. See Multiplicity in exploratory studies, below, for further discussion of proposed endotypes.

Finally, identifying which individuals respond best to preventive interventions is an important but difficult goal. In the scenario depicted in Fig. 2c (underlying heterogeneity in both risk of progression and preventive treatment effect among participants) the preventive effect (HR) differs among individuals, yet the average effect is similar to that in the scenario shown in Fig. 2a (all participants have identical underlying risk and treatment effect is identical). Clearly, the pattern and magnitude of heterogeneity matter. There are countless possible scenarios, including ones in which subgroups have increased risk when treated. An effect modifier does not necessarily need to influence the risk (such a scenario can also give similar results, although this was not shown in Fig. 2). Individual preventive effects can never be directly observed but heterogeneity in treatment effects can be examined in subgroups. This introduces multiplicity and low statistical power. Trials are typically not powered to detect subgroup effects [7] and the required sample size for robust identification of effect heterogeneity is approximately four times larger than for finding overall effect in some scenarios (Table 1). Moreover, treatment effects can vary over time (Fig. 2d). Robust identification of time-varying treatment effects also requires a very large sample size. An additional complicating factor is that identifying treatment effect heterogeneity may depend on the type of efficacy measure used. Different conclusions may arise depending on whether the effect measure is a ratio (such as the HR) or a difference (such as the risk difference or survival difference). While identifying treatment effect heterogeneity is clearly important, we should temper our expectations about its exploration.

**Table 1:** Practical aspects of different types of type 1 diabetes prevention or interception trial designs in populations at increasing risk

Practical aspect	General population children	Genetically susceptible children (without IA)	People with IA (stage 1 disease)	People with IA and dysglycaemia (stage 2 disease)
Identification of eligible individuals	Start early in life	Start early, ~1–10% of population eligible	Requires screening for IA with confirmation	Requires both screening for IA and multiple repeated testing for glycaemia [11]
Examples of interventions, ongoing or published	None	Hydrolysed infant formula [15], oral insulin [41], probiotics [42], SARS-CoV-2 vaccine [43]	Oral insulin [44], nicotinamide [45], GAD-alum [46]	Teplizumab [11]
Examples of potential future interventions	Vaccines (e.g. coxsackievirus [47]), nutritional interventions	Coxsackievirus vaccines [47]	Repurposed immunomodulatory drugs, possibly combinations	Repurposed immunomodulatory drugs, physical activity/lifestyle
Minimum required duration of follow-up	If outcome IA: ~4–6 years If outcome T1D: ~10–12 years	If outcome IA: ~4–6 years If outcome T1D: ~10–12 years	Median time to clinical T1D at least ~5–6 years (>20 years for some)	Median time to clinical T1D ~2–3 years (>8 years for some)
Assumed cumulative risk of endpoint during follow-up in the placebo group^a^	0.3%	10%	20%	50%
Sample size for 85% power if efficacy is 50% / 30%^b^	48,000/145,000	1450 / 4500	540 / 1800	200 / 650
Sample size for 85% power to detect heterogeneous effect if half of the participants are strong responders (66% efficacy) and rest are weak responders (33% efficacy)^b^	175,000	5675	2750	1000
Sample size for 2 × 2 factorial trial to test two interventions, each with 30% efficacy, assuming additivity of log HRs^c^	165,000	5,000	2,400	900

^a^For power and sample size calculations, the number of events is essential. The percentages assumed here can arise from a relatively short follow-up in higher-risk population, or longer follow-up of a comparably lower-risk population within each category of the target population

^b^Minimum required sample size based on simulations, 1:1 allocation of intervention, complete compliance and no loss to follow-up. Efficacy = (1−HR) × 100. Repeated simulations 1000–2000 times with constant and proportion hazards, two-sided tests called significant if *p*<0.05, unless accounting for three tests: *p*<0.0167 (0.05/3), for trials with three outcomes (e.g. type 1 diabetes, coeliac disease and infectious disease hospitalisation in general population primary prevention trials, see section 'The case for an antiviral vaccine trial with multiple outcomes’ for further elaboration). Tests for intervention-by-effect-modifier interaction (to detect heterogeneous effect) were called significant if *p*_interaction_ <0.025, since the interaction test is in addition to the overall test of efficacy. Example code for simulations is shown in the ESM Methods section

^c^Randomly allocate participants 1:1:1:1 to placebo, A only, B only, or A and B. Assume no interaction in the Cox model, meaning the HR for those with A and B compared with placebo is the product of the two main effects, here 0.7 × 0.7 = 0.49. While it is assumed here that the potential interaction (synergy or antagonism) is not of primary interest, several scenarios with twofold stronger or weaker effects of one factor in the presence of the other gave around 70% or higher power to detect significant interaction at *p*_interaction_ <0.025 with the listed sample size

IA, islet autoimmunity; T1D, type 1 diabetes

### Multiplicity

Investigators wish to extract as much knowledge as possible from their studies but multiplicity comes at a cost. Multiple testing increases the probability of chance findings, while accounting for multiple testing in the statistical analysis reduced statistical power. The number of comparisons is the product of the number of outcomes, treatment variations, subgroups and time points investigated [16]. This is often underreported.

#### Multiplicity in pivotal trials

Subgroup analyses in trials are usually prespecified but investigators rarely prespecify the expected pattern of effects in subgroups. After the primary publication, additional exploratory analyses are often conducted, frequently without accounting for multiplicity. Humans have an inherent ability to recognise patterns even when none exist. If multiple exploratory analyses reveal a subgroup with seemingly better treatment effect, it becomes very difficult to disregard it. In the Diabetes Prevention Trial with oral insulin, prudent investigators followed up a plausible subgroup signal [38] with a new trial, and this showed no significant effect. While some have argued for more mechanistically oriented trials with careful selection of participants, this can also be seen as a rationale for minimising subgroup analyses. Heterogeneities in treatment effects discussed in this paragraph are related to features defined at baseline [39] but phenotypic heterogeneity may also appear during follow-up.

#### Multiplicity in exploratory studies, including analysis of proposed endotypes

**Fig. 3:**
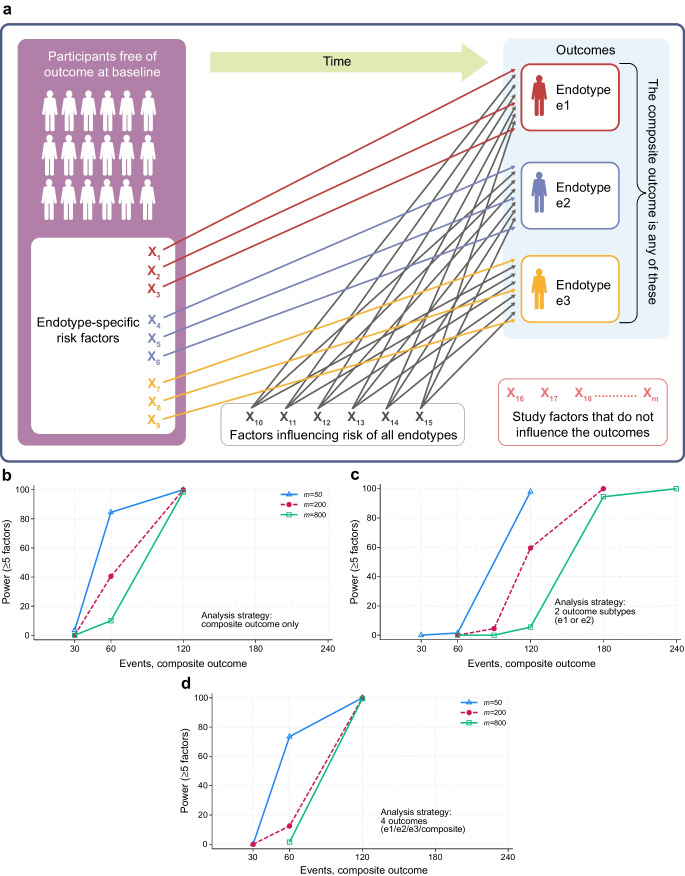
Statistical power for different approaches to analysis of potential risk factors for type 1 diabetes endotypes. Endotypes are defined here as subsets of type-1-diabetes-related phenotypes that have partially unique risk factors or causes. (**a**) Illustration of the underlying scenario for all analyses, involving three equally common and mutually exclusive endotypes (e.g. based on composition of islet autoantibodies or other measurable biomarker or phenotype). Out of a total of 15 true risk factors (X_1 _– X_15_), three sets of three risk factors are unique to each of the three endotypes (coloured maroon, purple and ochre), while six others affect all endotypes alike. None of the other factors studied affect the outcomes. The total number of potential risk factors studied, *m*, is 50, 200 or 800. The *m* factors studied as potential risk factors are all continuous standard normal variables (mean 0, SD 1), and independent of each other. Each risk factor affects the outcome with an HR of 2.0 per SD increase, a strong effect. The study design is a prospective case–control study nested within a birth cohort where cases are selected after end of follow-up with twice as many controls as cases, randomly selected. (**b**–**d**) Results of simulations to estimate statistical power to detect five or more of the 15 true, strong risk factors in three different analysis strategies for investigating *m* potential risk factors when these endotypes have been defined and measured during or at the end of follow-up. (**b**) Analysis of only one endpoint: composite endpoint only (any of e1, e2 or e3). (**c**) Analysis of two outcomes: endotypes 1 and 2 only. (**d**) Analysis of four outcomes: each of the three endotypes e1, e2 and e3 plus the composite. All strategies control the false discovery rate at <0.05 (Benjamini–Hochberg procedure), correcting for *o* × *m* tests, where *o* is the number of outcomes tested. The graphs show the power (probability, %) to detect at least five of the 15 true risk factors. For additional details of the simulated scenarios, see ESM Methods; additional aspects of power and false discoveries are shown ESM Figs 2 and 3

Type 1 diabetes prevention trials, and even the largest birth cohorts, have limited sample size for exploration. Handling of multiplicity becomes more complex when investigating subgroups of type-1-diabetes-related phenotypes (e.g. defined by islet autoantibodies or age at diagnosis of type 1 diabetes). Such subgroups are sometimes proposed to be endotypes. The endotype concept is somewhat elusive in practice, as further elaborated in ESM Methods. In the current review, I define endotypes as subsets of type-1-diabetes-related phenotypes that have at least partially unique causal risk factors. For omics and other exploratory studies, it is crucial to steward the data from prospective studies and trials responsibly. If endotype-specific risk factors do not exist, attempting to explore them leads to loss of statistical power and/or an increase in false positives. If they do exist, investigating endotype-specific associations clearly increases the chance of detecting such associations but still at the cost of increased multiplicity. The optimal analysis strategy is not intuitive. Again, numerous scenarios are conceivable but I have simulated a simple one to assess the performance of different analysis strategies (see Fig. 3). Figure 3a illustrates the conceptualisation of endotypes used in the simulations, where some risk factors are endotype-specific and others affect all endotypes equally. Each of 15 true risk factors has a strong effect and is explored among a larger set of potential risk factors that have no effect.

Figure 3b–d show statistical power to detect at least five of the 15 true risk factors when controlling the false discovery rate at <5%. The study design was case–control nested within a birth cohort with cases selected at the end of follow-up and twice as many controls as cases. The simulated scenario assumes very strong effects of each risk factor (an HR of 2.0 per SD increase). The simulation results show that even with such strong effect, studies with fewer than 100 cases are unlikely to be informative if more than 50 potential factors are studied in this scenario. Figure 3 also illustrates that in this setting, the practice of analysing two proposed endotypes such as IAA first and GADA first, while ignoring or not being able to infer other potential endotypes (Fig. 3c), gives weaker power overall compared with the strategy of analysing the composite outcome only (Fig. 3b). The ability to identify a signal for the two selected proposed endotypes has been lost due to noise of multiple testing with limited statistical power. When the number of individuals developing type 1 diabetes during follow-up is around 200, the expected number of true risk factors reliably identified is approximately 10–14 out of the 15, across the analysis strategies (ESM Fig. 2). In contrast, ignoring multiplicity results in false discovery rates >40% if investigating 200 factors and >75% if investigating 800 factors (ESM Fig. 3; details of the simulations are given in ESM Methods). In the real world, there may be much weaker effects than those simulated here, non-linear effects and measurement errors, that may drastically lower the statistical power (as well as confounding or other biases, which may distort results). The many cohort studies with less than 100 events using omics, or even multi-omics (typically >1000 potential risk factors studied), should be interpreted with great caution unless rigorously replicated in independent studies [40].

## Types of trials and pragmatism

Type of prevention trial can be grouped by the risk of clinical type 1 diabetes in the target population, which partially corresponds to disease stage. The required trial size is strongly influenced by the number of individuals expected to experience the primary outcome and assumed efficacy of the intervention (Table 1). Most type 1 diabetes prevention or interception trials to date have included individuals at high risk with intensive follow-up [7, 13]. While requiring moderate sample size, the time and resources needed to recruit and follow participants are daunting.

There is an inherent conflict between minimising the number of trial participants exposed to interventions with an uncertain effect and potential harm, and the necessity of exposing many people to an approved intervention when the evidence for its efficacy and safety is uncertain, particularly when based on a single trial with a small sample size.

Pragmatism comes into play when deciding whether to initiate a trial. The TRIGR investigators argued that, despite the weaknesses of the observational studies on this topic, the importance of infant feeding and its potential implications justified conducting a primary prevention trial [15]. A similar argument can probably be used for many other factors: the disagreement over existing observational studies may never be solved without randomised trial evidence. Steps have been taken towards a vaccine trial to prevent infections with potentially diabetogenic viruses despite conflicting evidence for their role in the aetiology of type 1 diabetes [48]. Primary prevention trials have been initiated involving oral insulin, probiotics [41, 42] and SARS-CoV-2 vaccination [43] as part of the Global Platform for the Prevention of Autoimmune Diabetes (GPPAD).

Trials can be designed with varying degrees of pragmatism, ranging from mechanistic to pragmatic [49, 50]. Mechanistic trials aim to learn about mechanisms of action, while pragmatic trials minimise intensive follow-up and collection of biomarkers, relying instead on existing infrastructure, such as electronic health records, to reduce the cost per participant and allow for the inclusion of much larger numbers of participants. To put it bluntly, pragmatic trialists prioritise determining whether an intervention works over understanding why or how it works.

Primary prevention trials target people who are not patients. The general population, especially children, are typically not in contact with the healthcare system except for routine preventive measures such as childhood vaccination. Vaccine trials provide a useful analogy for primary type 1 diabetes prevention trials. Many vaccine-preventable diseases are rare, severe outcomes of common infections, and it is difficult to predict who will experience a severe outcome. By intervening in the entire population, the incidence of severe disease in the population can be reduced.

### The case for an antiviral vaccine trial with multiple outcomes

One pragmatic approach to initiate a sufficiently large trial for primary prevention of type 1 diabetes is to collaborate with other fields to assess the impact of a multivalent antiviral vaccine on infectious disease and possibly other outcomes, in addition to type 1 diabetes. The likelihood of health benefits beyond reduced diabetes risk could make such an intervention more appealing and cost effective. Enterovirus infections are common and often asymptomatic but can lead to serious conditions such as meningitis, myocarditis and flaccid paralysis [47]. Vaccines targeting enterovirus 71 are licenced in China for the prevention of hand, foot and mouth disease [51] and have been combined with a hepatitis B vaccine in a single shot [52]. The pivotal trial of the BNT162b2 mRNA SARS-CoV-2 vaccine to prevent severe COVID-19 was based on over 40,000 adults, while the trial targeting children aged 6–23 months included less than 2000 individuals, with only a small subset aged 6 months [53]. Although SARS-CoV-2 vaccination of children aged 6 months or older is approved, it is not generally recommended in many countries, creating an opportunity for a placebo-controlled trial. Such studies could in principle be done in the general population, requiring inclusion of well over 40,000 participants, or in a cohort at genetically high risk for type 1 diabetes, reducing the sample size to fewer than 5000 (Table 1). Note that the efficacy assumed for the sample size requirements for a vaccine trial to prevent type 1 diabetes reflects a total effect that can be due to a range of scenarios: from complete prevention of the viruses where 30% (or 50%) of type 1 diabetes is due to these infections, to 50% (or 30%) prevention of virus infections where 100% of type 1 diabetes is due to these virus infections occurring early in life, as underlying, necessary factors in the aetiology. These may of course still be optimistic efficacy assumptions. Randomised trials of vaccines against SARS-CoV-2 and of multiple enteroviruses could provide critical insight into their potential to prevent severe outcomes of these infections, while at the same time testing the efficacy for type 1 diabetes prevention even if their role in type 1 diabetes aetiology remain uncertain.

## Summary and discussion

Questions remain about the best path forward to achieve the goal of preventing type 1 diabetes. I have sought to provide alternative perspectives on prevention strategies and the influence of heterogeneity and multiplicity using simulations. While the simulated scenarios are simplified, they offer valuable insight. Heterogeneity is an inherent feature of complex human diseases and it will likely never be fully controlled. Battaglia et al defined an endotype as a subtype of type 1 diabetes identifiable by a distinct functional or pathobiological mechanism that is also tractable therapeutically [33]. The conceptualisation of endotypes in the current review (Fig. 3a) differs somewhat from the latter definition. In the interest of primary prevention, we should seek to discover actionable risk factors that are not endotype-specific. This is because aetiological factors affecting all cases of type 1 diabetes are likely to be more efficacious in preventing (stage 3) type 1 diabetes.

A trial or intervention targeting a homogenous group of high-risk individuals would require extensive selection efforts and would apply only to a small proportion of those who will ultimately develop type 1 diabetes. Furthermore, homogeneity in risk does not guarantee greater efficacy of a preventive intervention. Conversely, implementing a population-wide infant vaccination programme poses significant challenges, not least the potential for adverse effects that could undermine public trust in the programme. Sample size simulations suggest that a primary prevention trial focused on genetically susceptible individuals may be the most feasible approach, although comprehensive total cost assessments remain essential. Population screening for islet autoantibodies offers a way to identify a large proportion of people who will get type 1 diabetes in the future but it may be difficult to reverse autoimmunity, and there are currently limited options for prevention for most screen-positive individuals, especially in countries where teplizumab is not yet approved. The current review has not considered potential harms inflicted by preventive interventions but these must be monitored in trials and in practice.

Innovative designs including 2 × 2 factorial designs and adaptive designs have been suggested for more rapid accumulation of evidence for prevention or interception of type 1 diabetes [54]. Such designs require careful planning and may be complex to interpret; obstacles have been discussed, with possible solutions [54]. Factorial designs are especially promising in terms of efficiency (illustrated in Table 1 for time-to-event outcomes). If two factors act additively or possibly even synergistically, two new treatments can be tested with a sample size only moderately larger than that of a single factor trial. However, primary or other prevention trials with time-to-event outcomes would still require a relatively large sample size. In general, the uncertainties in efficacy estimates discussed in the current review mainly apply to time-to-event outcomes and not necessarily to continuous outcomes such as C-peptide levels.

As for the question, raised in the section on judging trial evidence, of whether any future prevention trial should include teplizumab treatment, I cannot provide a definitive answer but I lean towards the position that it should not. There is now pressure from patient organisations and families to start using teplizumab for delaying progression to stage 3 disease in countries where it is currently not approved. In my view, a confirmation and more precise estimation of the efficacy of teplizumab to slow the progression from stage 2 to stage 3 type 1 diabetes in a phase III study would be reassuring, before deploying this treatment throughout the world. This could be done in a 2 × 2 factorial design simultaneously testing another intervention, possibly also demonstrating synergy.

## Supplementary Information

Below is the link to the electronic supplementary material.

ESM (PDF 818 KB)
