# Supplementary material for: Perspectives on prevention of type 1 diabetes and heterogeneities
Source: Diabetologia. 2025 Aug 6;68(10):2104–15. doi: 10.1007/s00125-025-06512-5 (PMC12423218; doi:10.1007/s00125-025-06512-5)
Supplement: Supplementary file 1 — ESM (PDF 818 KB) [file 125_2025_6512_MOESM1_ESM.pdf]

Electronic Supplementary Material (ESM)

Perspectives on prevention of type 1 diabetes and heterogeneities

Lars C. Stene, Norwegian Institute of Public Health, and Oslo Diabetes Research Centre
(lars.christian.stene@fhi.no)

Contents

ESM Methods text: 2

Stata code for simulating statistical power and sample size requirements presented in Table 1 2

Parameters of simulated scenarios in main figure 2 – the TN10 teplizumab trial. 2

Parameters of simulated scenarios and strategies b-d in main figure 3. 4

Notes on the concept of endotypes 5

ESM References 6

ESM Figure 1. Large sample simulation results of scenarios in main Figure 2 8

ESM Figure 2. Mean number of true risk factors identified as significant in simulated scenarios with 15 true risk factors by analysis strategy a-c (supplement to main Figure 3). 9

ESM Figure 3. Estimated false discovery rate by scenario if ignoring multiple testing. 10

ESM Methods text:

Stata code for simulating statistical power and sample size requirements presented in Table 1

Example code for scenario with a general population-based trial where 50% of the population have a feature making them good responders (66% efficacy, treatment effect hazard ratio 0.33), and the rest of the population are weaker responders to the preventive intervention (show efficacy of 33%; treatment hazard ratio of 0.67). This means that the marginal (overall) efficacy is the weighted average of the two efficacies: 0.5*66% + 0.5*33% = 50%, and that the interaction hazard ratio is 0.5 (0.33/0.66). Note that the overall proportion of events varies somewhat in the scenarios with heterogenous treatment effects from those with a homogeneous effect. Below is example code from one of the more complex scenarios with interaction (heterogeneous treatment effect), and simulated survival time data from an exponential survival time distribution (corresponding to constant hazard rates) and proportional hazards. Simulations were done with different sample size until power was approximately 85 %. Text after // (in green) in the code below is my explanatory comments.

```
clear all
global ln_hrA = ln(0.67) // set main effect (logHR) of treatment A (global macros retrieved with $ in front)
global ln_hrB = ln(1.0) // set main effect (logHR) of effect modifier B is zero (HR=1)
global ln_hr_int = ln(0.5) // set magnitude of interaction logHR between A and B
global lambda = 0.000036 // set baseline hazard rate for the placebo group
global   N = 200000 // set sample size
global rep = 1000 // set number of repetitions (reps) for simulations
matrix A=J($rep ,1,0) // make matrix in which to store results from each repeated simulation below
matrix colnames A= "pvalue"
forvalues i=1(1)$rep {
clear
qui: set obs $N // set sample size (number of observations) in each repeated trial
qui: gen A = rbinomial(1, 0.5) // 50% assigned intervention A
qui: gen B = rbinomial(1, 0.5) // 50% of the population have feature B which modifies the effect of intervention
qui: gen ABint = A*B // generate the interaction term
qui: gen ue1 = runiform() // this and next 4 lines simulate survival times under the given scenario
qui: gen stime_e1 = -log(ue1) / ($lambda * exp(A*$ln_hrA + B*$ln_hrB + ABint*$ln_hr_int ) )
qui: gen      fail_e1 = (stime_e1 < 84)
qui: replace stime_e1 = min(stime_e1, 84) // administrative censoring at 84 months, explain in the next section
qui: stset   stime_e1, failure(fail_e1) // Stata code to declare data to be survival data
qui: stcox A##B // run Cox regression with main effects and interaction between A and B
matrix A[`i',1]=2*normal(-abs(_b[1.A#1.B]/_se[1.A#1.B])) // store p-value of test for interaction in matrix A
} // loop end
clear
svmat A, names(col) // make data frame from matrix A of stored results. One line per simulated trial.
generate s2= (pvalue<0.05/2) // make indicator for each rep whether the test of interaction was significant at
p<0.025 (accounting for two tests, the main effect and the (current) tests of interaction.
collapse (mean) prop_s2 = s2 // collapse the data frame over all repetitions and keeps the mean of the binary
variable s2. This is the proportion of trials that were significant across all reps, which is the statistical power.
```

Parameters of simulated scenarios in main figure 2 – and additional comments on simulation results

Simulations were done in Stata v17 and 18, with example code given below. The simulations were done aiming to resemble as much as possible the original TN10 teplizumab trial. Constant hazard ratios were assumed (except in panel d), at a level resulting in approximately the same overall risk and proportion of T1D events as in the original TN10 Teplizumab trial (this assumption seems to fit reasonably well to the observed data and were also used in the original sample size estimations of the original trial [1]). A single run for each scenario were simulated, using set seed to ensure reproducibility. In addition, large sample simulations of the scenarios are shown in ESM Figure 1 to see large sample results with random variation essentially eliminated.

Panel **a**: Homogeneity: all individuals have constant and equal hazard and treatment effect is constant and equal for all individuals.

Penel **b** Heterogeneous risk: A standard normally distributed risk score (with mean zero, standard deviation 1) gives all individuals a different risk of progressing from stage 2 to stage 3 type 1 diabetes (T1D) (effect size HR

2.71 per unit, or standard deviation, increase). This variable is not related to treatment effect, so does not influence the treatment effect, which is instead assumed to be equal for all individuals. The risk factor is assumed unobserved and not accounted for in the analysis. Because of the high absolute risk of progression to T1D, and the so-called non-collapsibility of the hazard ratio, the true effect size is underestimated (biased towards the null value of HR = 1) because of the heterogeneous risk that is not accounted for. The true HR was set to 0.38, while the estimated, marginal large sample HR was 0.49. Also, for large absolute risks as here, the unobserved heterogeneity leads to a phenomenon where the most susceptible individuals are depleted early from the population under follow-up. This leads to an apparent falling hazard over time, more so in the placebo group. This gives the appearance of a diminishing treatment effect over time, and a quickly declining risk in the placebo group, even in the scenario where the true treatment effect is constant over time [2-4] (as illustrated in ESM Figure 1 e and g below).

Kaplan-Meier curves resembling the original TN10 trial was produced in scenarios where the true progression hazard is constant but appears to decline over time due to unobserved heterogeneity. The actual TN10 teplizumab trial included age and oral glucose tolerance test results at baseline as covariates to account for risk heterogeneity in the estimation of the efficacy hazard ratio in the Cox regression model, but the Kaplan-Meier curves from the original trial did not account for this heterogeneity. It is therefore possible that the apparent quick drop in survival in the early period after randomization in the placebo group at least potentially is explained by such risk heterogeneity rather than treatment effect heterogeneity or bias.

Panel **c** heterogeneous risk and treatment effect: Here the continuous risk score (as in panel b) also modifies the treatment effect with an interaction HR of 0.7 (treatment HR diminishes and efficacy increases with increasing levels of the unmeasured effect modifier). The treatment HR varies from an average of 0.66 for the lowest 15% of the distribution to 0.26 in the upper 15% of the distribution of the effect modifier. The true main effect hazard ratio of the treatment is 0.38 and observed estimated HR for treatment effect is the marginal large sample HR across heterogeneous individuals, estimated to 0.48. Similar results were also obtained when simulating a binary effect modifier present in 50% of the population such that this half had a preventive intervention efficacy of 66% while the other half had a 33% preventive intervention efficacy (results not shown).

Panel **d**: All individuals have equal baseline risk and equal treatment effect, but the treatment effect diminishes over time, equally for all individuals, from HR 0.15 at start of follow-up to 0.7 by 84 months.
The various estimates of efficacy shown in main Figure 2 were the hazard ratio (HR), the risk ratio (RR), defined as the ratio of the observed proportion progressing to T1D in the treatment group over that in the placebo group (the ratio of two incidence proportions, sometimes referred to as event rates, even though they are not rates). In addition, the difference between treatment arms in median survival time was estimated, with 95% confidence intervals shown in main Figure 2 based on bootstrap resampling with 1000 repetitions and taking the 2.5 and 97.5 percentiles as the lower and upper 95% confidence limits. An alternative efficacy measure which can be estimated even if fewer than 50% experience the endpoint during the follow-up period, and which takes the whole survival curve into account, is the difference in restricted mean survival time, RMST [5-7]. It is restricted to the longest observed survival time, here set to 84 months in the simulations. If everybody had experienced the outcome during follow-up, the mean survival time would have been the area under the survival curve. The restricted mean survival time is instead the area under the survival curve up to the longest observed survival time. In the simulations, I applied so called administrative censoring at the end of follow-up at 84 months and additional uniformly distributed (uninformative) censoring to simulate the consecutive recruitment of participants or drop-outs and hence variation in follow-up time at the end of study to attain roughly the same proportion of events (T1D during follow-up) as in the TN10 teplizumab trial. Panel f was created with predictions from flexible parametric survival models (Stata plug in program stpm3), modelling the hazard using splines [8, 9].

Code for simulating scenario a (homogeneity) in Stata software

1. First install the user written programmes

survsim [10-12] by running “ssc install survsim”. It is also possible to simulate with standard Stata code as explained by Crowther in the cited references, and also done in the example in the previous section for power and sample size simulations. survsim is especially convenient for simulating complex survival times.

rmst2 [7]. Restricted Mean Survival Time (RMST) is also provided in the standard Stata software and other software, but rmst2 is convenient in how it provides 95% confidence intervals for differences in RMST.

2. Simulate survival data with censoring

```
set obs 76
set seed 30971
gen trt = rbinomial(1,0.5)
global trt_hr=0.5
global trt_loghr = ln($trt_hr)
survsim stime1 , distr(exp) lambda(0.025) covariates(trt $trt_loghr) // generates survival times from a exponential survival distribution (constant hazard rates) with baseline hazard rate of 0.025, and proportional hazards with effect size HR=0.50. (baseline hazards in scenarios b, c and d were 0.030, 0.035 and 0.025)
gen sens=runiform(6, 120) // generate uniform censoring times from time 6 months, up to 120 months.
gen stime2=min(stime,sens) // replace survival time with censoring time if it occurred before event time
replace stime2=min(stime2, 84) // administrative censoring at 84 mo (end of follow-up)
tostring stime1, gen(stime1_str2dp) format(%6.2f) force // trick to force rounding to 2 decimal places
tostring stime2, gen(stime2_str2dp) format(%6.2f) force
gen event=(stime1_str2dp==stime2_str2dp & stime1<84)
```

3. Analyse the simulated data

```
stset stime2, f(event) // Stata code to declare data to be survival data before analysis
stcox trt // Cox regression to estimate hazard ratio (HR)
binreg _d trt, rr // binomial log-linear regression to estimate risk ratio (RR), formally incidence proportion ratio
strmst2 trt, tau(84) rmtl // estimates difference in restricted mean survival at 84 months
*
cap program drop median_diff
program define median_diff, rclass // program to store point estimates for median survival in each trt group
 stci if trt==0, median
 local m0 = r(p50)
 stci if trt==1, median
 local m1 = r(p50)
  return  scalar diff_median= `m1' - `m0'
 end
bootstrap r(diff_median), reps(1000) seed(123): median_diff // running the programme on bootstrap samples with 1000 reps to retrieve 95% confidence interval as the 2.5th and 97.5th percentiles. Around 2-5% of bootstrap samples failed to produce an estimate of the difference in median survival times because at the estimated survival at the end of follow-up was more than 50% in at least one of the treatment arms, an inbuilt weakness of the median survival time as discussed in the main text.
sts graph, by(trt) // plot Kaplan-Meier survival curves
```

Parameters of simulated scenarios and strategies b-d in main figure 3.

Scenario with multiple risk factors and endotypes

The simulations were set up to evaluate the impact on statistical power to find true associations with different strategies for handling possible endotypes (type 1 diabetes-related disease outcomes) and handling of multiplicity of tests. The simulated studies tested *m* (independent) potential predictors for association with outcome, simulating scenarios with *m*=50, 200 or 800. Of the *m* potential predictors, 15 were true risk factors influencing the outcome with a hazard ratio (HR)=2.0 (a strong effect, see more below), and the remaining predictors had no effect on outcome. Outcome (islet autoimmunity, type 1 diabetes) is subdivided in 3 mutually exclusive endotypes e1, e2 and e3 (can be thought of as e.g. insulin autoantibodies first, GAD antibodies first, and others, or alternatively as 3 groups of age at diagnosis of type 1 diabetes). Of the 15 true risk factors, three (x_1, x_2, x_3) affect only e1, three others (x_4, x_5, x_6) affect only e2, while yet three others (x_7, x_8, x_9) affect only e3, and the six remaining factors (x_{10}-x_{15}) affect all endotypes. Each of 3 endotypes are equally common. The observed HR for a risk factor that affects only one endotype, if tested for association with the composite outcome will then be 1.26 [$e^{(1/3)}$ x ln(HR)]. A cohort size of 30,000 was simulated with a 3% absolute risk of the composite outcome after end of follow-up (~900 events of composite outcome). Cases and twice as many controls as cases were selected randomly from the cohort (varying sample size for the different scenarios with varying number with outcome) and analysed with univariable logistic regression for 200 simulations of each scenario. (note that because of the so-called non-collapsibility of the hazard ratio and the odds ratio, univariable regression models will observe a slightly weaker hazard ratio or odds ratio, approximately 1.9). Simulations assumed a baseline hazard rate (for those with zero for all potential risk factors) of 12 per 100,000 per year for any outcome, corresponding to a 3% absolute risk by 12 years of age, similar to high-risk cohorts with first

degree relatives of patients with type 1 diabetes or carriers of susceptible HLA genotypes. The outcome subtypes (endotypes) were defined as competing events, as for insulin-autoantibodies first (e1), GAD-autoantibodies first (e2) and the rests (e3) or subtypes by age-at onset of outcome.

The simulated scenarios involve risk factors with relatively strong effects. As an example from other fields, the well-established relation between blood cholesterol and risk of ischemic heart disease mortality showed a pooled hazard ratio of approximately 2 per standard deviation increase among 40–49-year-old people, and weaker associations at older ages, in a large meta-analysis, after adjusting for regression dilution bias [13]. With the background hazard and risk factors simulated in the current paper, the area under the ROC-curve (AUROC) for the true model with all risk factors correctly modelled is approximately 0.90, and the AUROC for each single true risk factor is approximately 0.67 (for a single endotype-specific risk factor investigated for association with the composite outcome only, the AUROC is approximately 0.54). AUROC was not estimated in the simulations because the current focus is on identification of actionable risk factors for primary prevention of islet autoimmunity/type 1 diabetes, not on prediction or discrimination.

Analysis strategies
Analysis strategies with respect to outcome subgroups investigated in main Figure 3, panels **b**, **c**, and **d** correspond to panels **a**, **b**, **c** in ESM Figures 2 and 3: All of *m* potential risk factors are analysed in all strategies, and p-values were corrected for multiplicity using the Benjamini-Hochberg procedure to control the false discovery rate at <0.05 in all strategies. The number of tests is *o* x *m*, where *o* is the number of outcomes (endotypes) tested. Strategy **a**: analyse only the composite outcome (any of the sub-types e1, e2 or e3). Correcting p-values for *m* tests. Strategy **b**: investigate two outcome sub-groups e1 and e2, correcting for *m* x 2 tests. This strategy will never identify the three risk factors that affect only e3, so only the 12 others that affect either e1, e2 or all endotypes can be identified. Strategy **c**: investigate each of three subtypes of outcomes plus the composite outcome. *m* x 4 tests were accounted for.

Notes on the concept of endotypes

There are many definitions and conceptualisations of endotypes in the literature. Any practical application involves the measurement or observation of one or more phenotypic traits (measurable characteristic or trait of an individual). The concept is tightly related to some aspects of heterogeneity discussed in the main text. Below, I first show a few published proposed definitions of endotype, before elaborating on practical difficulties.

Battaglia et al Diabetes Care 2020 [14]:

> *a subtype of type 1 diabetes identifiable by a distinct functional or pathobiological mechanism that is also tractable therapeutically*

Redondo and Morgan, Nat Rev Endocrionl 2023 [15]:

> *disease subtypes each of which has a unique aetiopathogenesis that is amenable to a particular intervention, will help apply precision medicine to T1DM*

Anderson, Lancet 2008 (on asthma endotypes) [16]:

> *Endotype—a contraction of endophenotype—is a subtype of disease defined functionally and pathologically by a molecular mechanism or by treatment response*

The Wikipedia entry on endotype (https://en.wikipedia.org/wiki/Endotype ; accessed November 2019, and again May 28, 2025 to confirm it was not changed):

> *An endotype is a subtype of a condition, which is defined by a distinct functional or pathobiological mechanism*

Most of these definitions encompass both aetiological mechanisms *and* treatment responses or pathways, which in practice may not necessarily overlap. Mechanisms involved in early-stage disease such as initiation of islet autoimmunity, may not necessarily directly reflect pathogenetic pathways activated late in the disease process

such as those involved in beta-cell killing by cytotoxic T-cells. The first may be most relevant for primary prevention and the latter most relevant for interception in late-stage disease or treatment in stage 3 disease.

In the end, all practical use of the endotype concept relies on measuring or observing some phenotypic trait [15, 17-20]. One can make attempts to correlate these with biomarkers thought to reflect a mechanistic pathway, to a genetic variant or even treatment response, but in practice causality can rarely be proven. The exception would be in a randomized experiment where an effect is observed in a pre-determined sub-group based on phenotypic characteristics and not in other subgroups. The field have struggled to identify well-established non-genetic aetiological factor for type 1 diabetes. Whether subdividing study populations by proposed endotypes will aid or harm identification of causal factors in primary prevention or late-stage interception remains an open question. The simulations shown in Figure 3 argues that in at least one scenario, the attempt to define endotypes is likely to complicate matters. Merely describing phenotypic differences by for instance age at diagnosis can be due to underlying age-effects rather than true endotypes in the above defined sense. In practice, most proposed endotypes will remain proposed endotypes. It is essentially impossible to prove in operational terms, and will in practice result in potentially endless number of subtypes based on phenotypes proposed to represent different pathogenic mechanism.

The definition used in the simulations showed in this review focus on aetiological factors (factors that causally influence the risk of disease). With words, the definition of endotypes illustrated in main Figure 3, panel a is:

subsets type 1 diabetes-related phenotypes that have partially unique causal risk factors

This definition is in line with for instance Virgin & Todd, Cell 2010 [21], and traces back at least to Rothman Am J Epidemiol 1976 [22]. Rothman, however, proposed that in general, the “endotypes” represent the same multifactorial disease resulting from different subsets of causes, or risk factors. The simulations in main Figure 3 investigated the implications for data analysis in the hypothetical scenario that endotypes both exist, and that they can be perfectly measured to correctly classify individuals. Again, in practice, even if such endotypes exist, the correlation between measurable phenotypes (or proxies for it) and true endotypes is likely to be imperfect and hence lead to weakening of any observed statistical association between risk factors and proposed endotypes. As shown in the simulations in main Figure 3, the necessary correction for multiplicity correction when proposing and analysing many endotypes will further reduce the robustness and applicability of the endotype concept.

ESM References

[1] Herold KC, Bundy BN, Long SA, et al. (2019) An anti-CD3 antibody, Teplizumab, in relatives at risk for type 1 diabetes. N Engl J Med 381: 603-613

[2] Gomes MGM, Gordon SB, Lalloo DG (2016) Clinical trials: The mathematics of falling vaccine efficacy with rising disease incidence. Vaccine 34: 3007-3009

[3] Stensrud MJ, Valberg M, Roysland K, Aalen OO (2017) Exploring Selection Bias by Causal Frailty Models: The Magnitude Matters. Epidemiology 28: 379-386

[4] Stensrud MJ, Hernan MA (2020) Why test for proportional hazards? JAMA 323: 1401-1402

[5] Royston P, Parmar MK (2011) The use of restricted mean survival time to estimate the treatment effect in randomized clinical trials when the proportional hazards assumption is in doubt. Stat Med 30: 2409-2421

[6] Zhao L, Claggett B, Tian L, et al. (2016) On the restricted mean survival time curve in survival analysis. Biometrics 72: 215-221

[7] Cronin A, Tian L, Uno H (2016) strmst2 and strmst2pw: New commands to compare survival curves using the restricted mean survival time. Stata Journal 16: 702-716

[8] Lambert PC, Royston P (2009) Further development of flexible parametric models for survival analysis. Stata Journal 9: 265-290

[9] Royston P, Lambert PC (2011) Flexible parametric survival analysis using stata: beyond the Cox model. Stata Press, College Station, Texas

[10] Crowther MJ, Lambert PC (2012) Simulating complex survival data. Stata J 12: 674-687

[11] Crowther MJ, Lambert PC (2013) Simulating biologically plausible complex survival data. Stat Med 32: 4118-4134

[12] Crowther MJ (2022) Simulating time-to-event data from parametric distributions, custom distributions, competing-risks models, and general multistate models. Stata Journal 22: 3-24
[13] Prospective Studies Collaboration (2007) Blood cholesterol and vascular mortality by age, sex, and blood pressure: a meta-analysis of individual data from 61 prospective studies with 55,000 vascular deaths. Lancet 370: 1829-1839
[14] Battaglia M, Ahmed S, Anderson MS, et al. (2020) Introducing the endotype concept to address the challenge of disease heterogeneity in type 1 diabetes. Diabetes Care 43: 5-12
[15] Redondo MJ, Morgan NG (2023) Heterogeneity and endotypes in type 1 diabetes mellitus. Nat Rev Endocrinol 19: 542-554
[16] Anderson GP (2008) Endotyping asthma: new insights into key pathogenic mechanisms in a complex, heterogeneous disease. Lancet 372: 1107-1119
[17] Lötvall J, Akdis CA, Bacharier LB, et al. (2011) Asthma endotypes: a new approach to classification of disease entities within the asthma syndrome. J Allergy Clin Immunol 127: 355-360
[18] Bisgaard H, Pipper CB, Bønnelykke K (2011) Endotyping early childhood asthma by quantitative symptom assessment. J Allergy Clin Immunol 127: 1155-1164 e1152
[19] Leete P, Oram RA, McDonald TJ, et al. (2020) Studies of insulin and proinsulin in pancreas and serum support the existence of aetiopathological endotypes of type 1 diabetes associated with age at diagnosis. Diabetologia 63: 1258-1267
[20] Ray A, Camiolo M, Fitzpatrick A, Gauthier M, Wenzel SE (2020) Are we meeting the promise of endotypes and precision medicine in asthma? Physiol Rev 100: 983-1017
[21] Virgin HW, Todd JA (2011) Metagenomics and personalized medicine. Cell 147: 44-56
[22] Rothman KJ (1976) Causes. Am J Epidemiol 104: 587-592

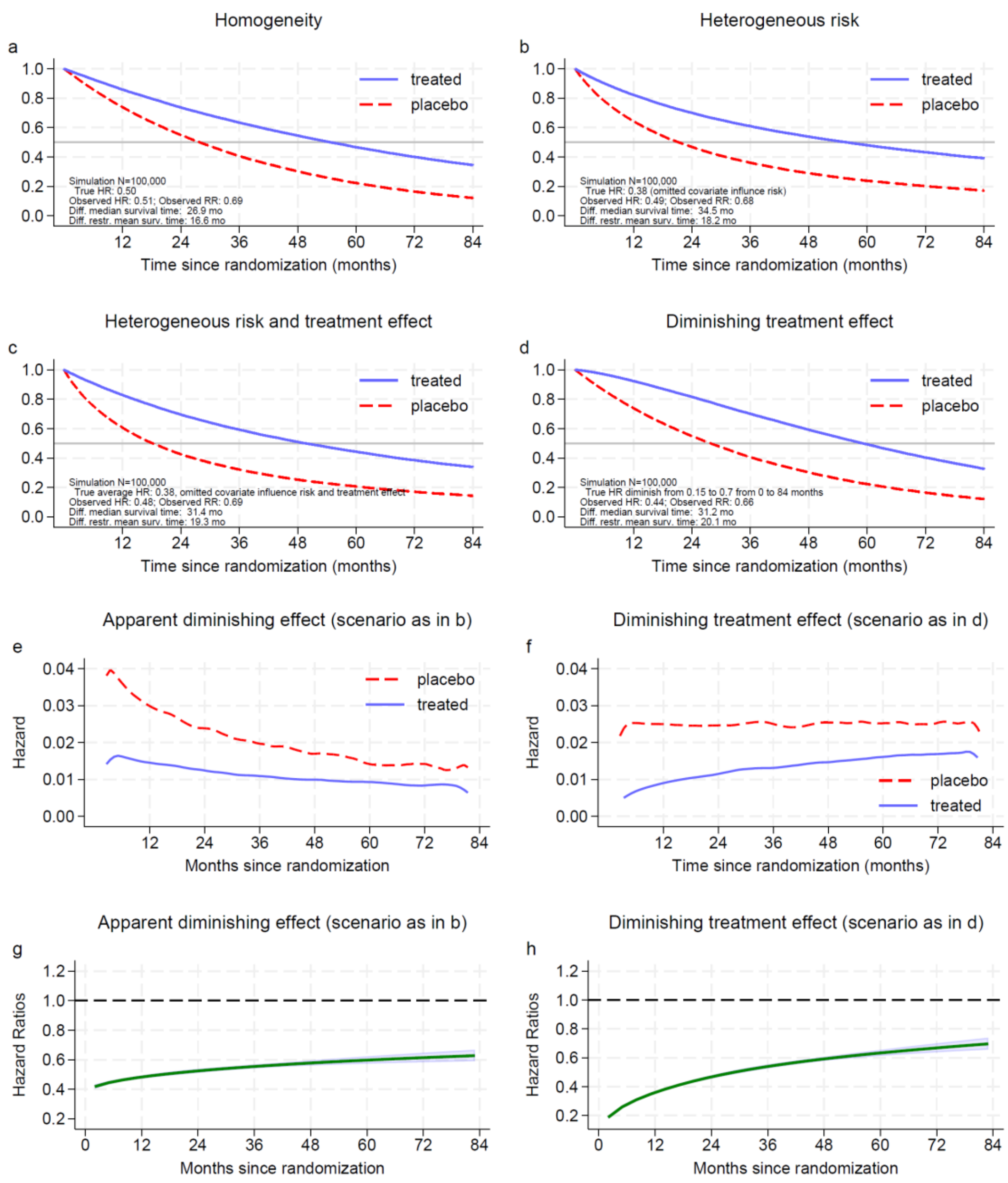

ESM Figure 1. Large sample simulation results of scenarios in main Figure 2.
Simulations of trials resembling the TN10 Teplizumab prevention trial with different underlying scenarios, for N=100 000 to minimize random variation. Panels **a-d** show survival probabilities as in main Figure 2.
a Homogeneity: all individuals have an equal hazard rate of progression to stage 3 type 1 diabetes, and the treatment effect is equal for all individuals, with a true hazard ratio (HR) of 0.50. **b**: Individuals have different levels of a risk factor, and hence heterogeneous risk of progressing to type 1 diabetes, but the treatment effect (HR) is equal (homogeneous) for all individuals. The risk factor is assumed unobserved and omitted from the analysis **c**: heterogeneous risk and treatment effect. The continuous risk score (as in panel b) also modifies the treatment effect with an interaction HR of 0.7. **d**: All individuals have equal baseline risk and equal treatment effect, but the treatment effect diminishes over time, equally for all individuals, from HR 0.15 at start of follow-up to 0.7 by 84 months. Additional details of the simulations are found in the ESM Methods text. Panels **e-f** show the (kernel smoothed) hazard *rates*, and panels **g-h** show estimated hazard ratios (effects) over time, allowing for time-varying effects. Panels **e** and **g** illustrate the *apparent* diminishing effects of intervention that is entirely due to heterogeneity in risk that is not accounted for in the analysis (assumed unobserved, a risk factor is omitted from the model). This leads to a more pronounced depletion of susceptible individuals in the placebo group. Panels **f** and **h** shows the actual diminishing effect over time in a scenario as in d, where there is no heterogeneity in actual risk or treatment effect.

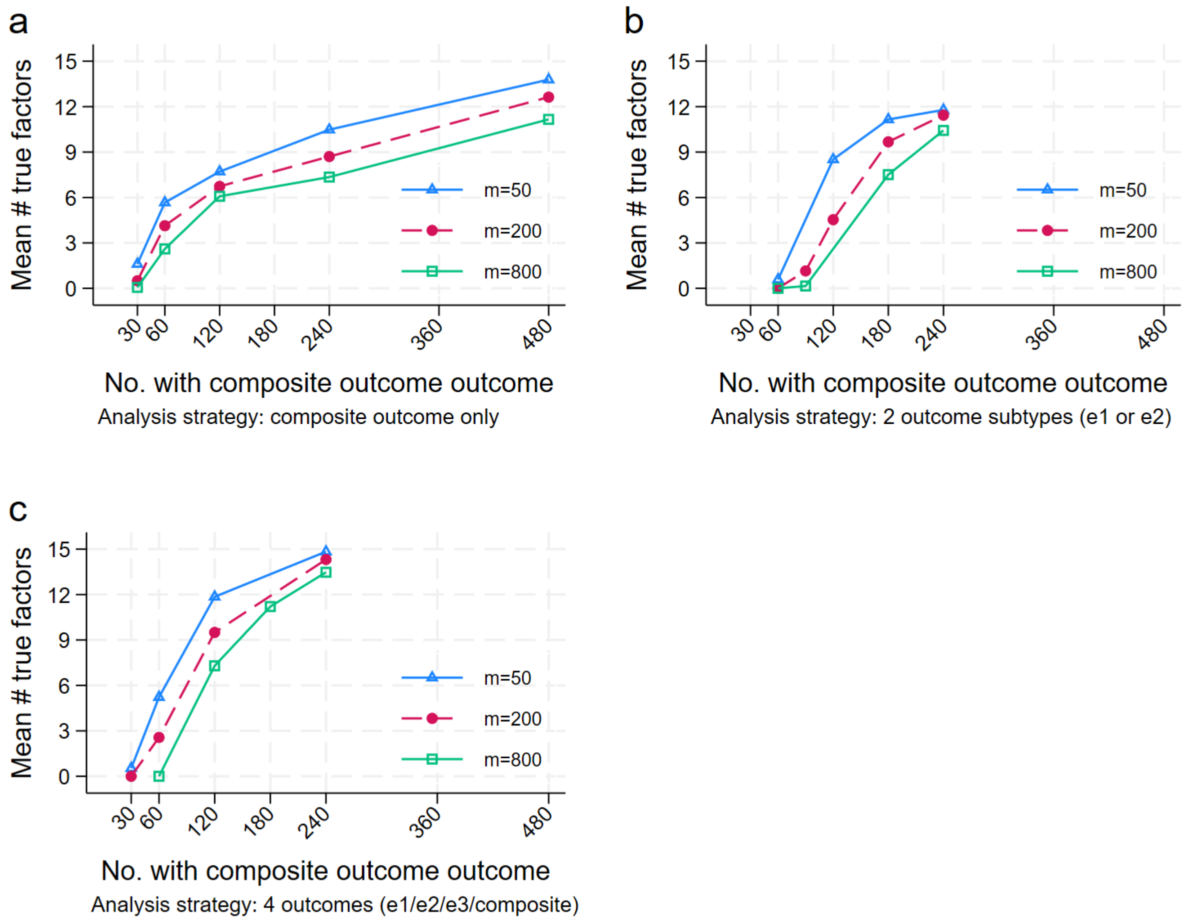

ESM Figure 2. Mean number of true risk factors identified as significant in simulated scenarios with 15 true risk factors by analysis strategy a-c (supplement to main Figure 3).

Details of strategies and simulated scenarios described in ESM Methods text. m = total number of potential risk factors tested for association with outcome. False discovery rate controlled at < 0.05 and accounting for m x o tests, where o is the number of outcomes investigated in each strategy (1 in **a**, 2 in **b** and 4 in **c**). Since three true risk factors affect only endotype e3, analysis strategy **b** (only investigating e1 and e2) can maximally identify 12 of the 15 true risk factors.

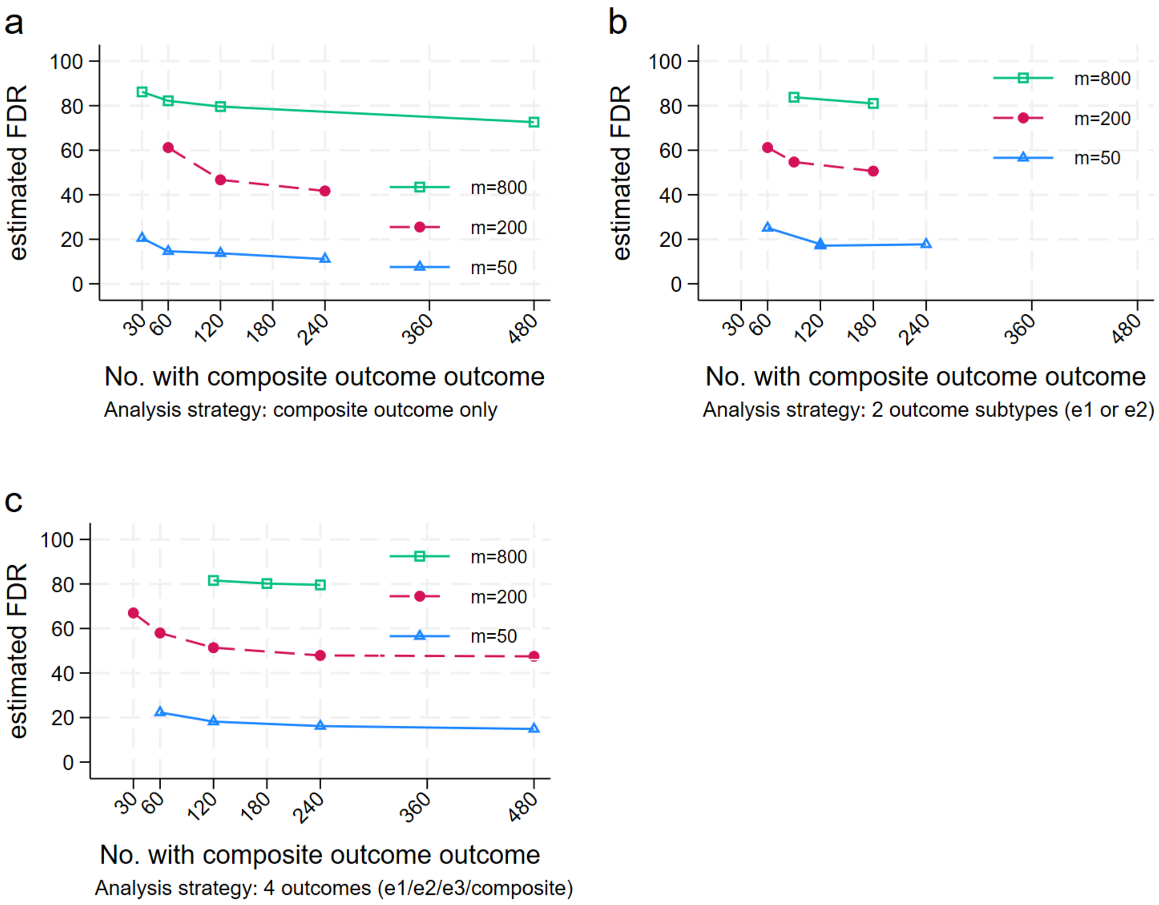

ESM Figure 3. Estimated false discovery rate by scenario if ignoring multiple testing.

Supplement to main Figure 3. Scenarios are described in Main Figure 3 with additional details in ESM Methods text. m=total number of potential risk factors studied. Number of tests done are *m* x *o*, where *o* is the number of outcomes investigated in each strategy (1 in **a**, 2 in **b** and 4 in **c**). Outcomes are three mutually exclusive and equally common subtypes of outcomes, termed endotypes e1, e2, and e3, as well as the composite consisting of any of the three subtypes. FDR: False discovery rate. Note that in this analysis, we inappropriately ignore multiple testing and call instead an association significant if the nominal (unadjusted) p-value is significant at the 5% level. Thus, FDR is not controlled at a certain level, but is estimated here to levels typically much higher than 5%.
